# Focus on proximal femur fractures: surgical options and outcome

**DOI:** 10.1007/s00068-020-01511-0

**Published:** 2020-10-28

**Authors:** Felix Bonnaire

**Affiliations:** Oberer Kreuzweg 4, 01097 Dresden, Germany

Dear Readers,

Proximal femur fractures are most common fractures in our elderly population and produce routine problems for trauma and orthopedic surgeons. It is common that they lead to immobility or hindrance in daily life. In addition, they can have surgical problems as well as multiple medical complications. For surgeons, it is important to know about both sides of the problem. It is significant that we know the fitness and comorbidities of patients and their risk factors for life and surgery.

We have several lessons learned in the last 10 years according to the surgery of unstable proximal femur fracture type 31A2.2–31A3.3 according the AO/OTA classification: short interval from injury to surgery is a positive factor; cephalo-medullary nails in different lengths and cerclages for unstable (sub-)trochanteric fractures are better for early mobilization in some fracture-types and have less complications than extramedullary devices as the DHS. In addition, details are important: the entry of the nail, varus position is not acceptable in any case, and the center–center position of the proximal part of the implant is the best position together with the small tip–apex index is almost a guarantee against the cut out.

The surgical problems are not the only ones: cardio-respiratory diseases, diabetes, too high and low body weight, renal insufficiency, anticoagulation and other medications need to be addressed as well as early mobilization, physical training, plan for rehabilitation and organization of future life circumstances of older people. Specialized clinical centers for geriatric surgery are instituted to improve these complex circumstances to avoid earlier or later readmission.

For these reasons, it is a good standard to control the own cases routinely for known and for new aspects. For example, what factors do exist to prevent readmission to the hospital, which is the most critical point for mortality?

In their paper “Predicting of readmissions in the first post-operative year following hip fracture surgery”, the authors collected demographic information, comorbidities, and in-hospital characteristics, as information regarding 1-year readmissions. Multivariate analysis of factors predictive of rehospitalizations was performed, followed by a logistic regression using all predictors with *p* < 0.05 and a predictive model for avoiding readmissions was created. The result is a very important information with consequences for surgeons and medical doctors at the same time [1].

The importance of new knowledge, implants and skills for avoiding complications can be visible in the study about “PFNA and DHS for AO/OTA 31-A fractures: radiographic measurements, morbidity and mortality”. In this report, two different implants, extramedullary and intramedullary, in different time zones (2006–2011 and 2012–2015), used to stabilize fractures according to AO/OTA 31-A2 classification were compared. In their retrospective study with 375 patients, they lost no patient in the follow-up for their inquired questions, which is very rare in similar reports. Using the PFNA for stabilizing these fractures, they report shorter operation times, less blood loss and less implant-related complications as cut-out, infections and failures. The comparison of TAD measurements, fracture reduction, central–central position and bad screw positions were significantly improved using the intramedullary implant. These factors improve the results together with the new implant. Details are interesting [2].

In the article “Reduced complication rates for unstable trochanteric fractures managed with third‐generation nails: Gamma 3 nail versus PFNA”, the clinical results of two different cephalo-medullary nails the third generation were compared without any influence of industrial partners [3]. All critical surgical knowledge and skills to avoid complications were analyzed and we saw that it is possible to avoid the typical “cut out” in a series of 106 patients with unstable trochanteric fractures type 31.A2 and A3 according to the AO/OTA classification. Intraoperative problems are seen and reported that could be avoided in the future generation implants and surgeons.

All the same, not all questions about the surgery on trochanteric fractures are answered. The discussion about the optimal length of the cephalo-medullary nail for unstable trochanteric fractures has not been ended. In their paper addressing this problem, ”Outcomes after unstable pertrochanteric femur fracture: intermediate versus long cephalomedullary nails”, the authors compare the results in these fractures for long and intermediate–long nails and found some advantages in the group of intermediate long nails without disadvantages in a retrospective study: an interesting study for further discussions and follow-up [4].

Another surgical detail is discussed by the authors of “The impact of cerclage cabling on unstable intertrochanteric and subtrochanteric femoral fractures: a retrospective review of 465 patients” [5]. The question whether a cerclage around the 31A2.2–31A3.3 fractures can help or not may produce more trouble. In this article, typical non-unions and infections are seen and analyzed. The discussion and the results are evident and interpretation will help the surgeon in his choice of using cerclages.

In summary, in this issue, *European Journal of Trauma and Emergency Surgery* addresses daily problems of surgeons and medical doctors with a big influence on patient mobility and mortality and financial challenges of national medical systems.
